# As Organised by and Worked at the Radcliffe Infirmary

**Published:** 1921-07-23

**Authors:** 


### THE OXFORD SCHEME.

### As Organised by and Worked at the Radcliffe Infirmary.

THE publication of the report of Lord Cave's Committee has brought the question of contributory schemes by the working-class for the upkeep of the voluntary hospitals prominently before the public. Of these, the Oxford Scheme is in one respect a pioneer, as it represents the first effort made to adapt a contributory scheme to the circumstances of a large agricultural district. Outside the City of Oxford the district served by the hospital covers the whole of Oxfordshire, large parts of Berkshire and Buckinghamshire, and a few villages in Gloucestershire and Northamptonshire, so that the extent of ground to be covered by the necessary organisation is large.

### CONTRIBUTIONS.

The scheme was originally founded on that which has proved so successful in Leicester, and after the election of a paid collector the Appeal Sub-Committee drew up the following clauses embodying the terms on which hospital benefit should be offered :

1. A contributor of 2d. a week is entitled to free treatment.
2. In the case of married people, when the husband and wife each subscribe 2d. a week their children are also entitled to free treatment up to wage-earning age, and from then until they are earning adult's wage, each child pays 1d. a week. The children of a widow or widower are also entitled to free treatment up to wage-earning age on the 2d. contributions of their parent.
3. Non-contributors will in future be expected to contribute towards their maintenance in the hospital, but no payment is required from old-age pensioners.
4. Every case must be accepted by a doctor or surgeon as suitable for hospital treatment.

Contributors to the hospital on these terms are no longer obliged to bring with them a subscriber's letter, as their payments constitute an insurance for hospital treatment.

Husbands and wives have one card, but as soon as a child starts wage-earning it has one of its own.

### URBAN AND RURAL ORGANISATION.

On these lines the Committee and collector quickly got to work. In the city the consent of the local trades unions was first obtained to the principle of a voluntary levy on the men's wages; the local firms were then circularised, and the proprietors and managers asked to place the scheme before their employees, and in many cases the collector obtained leave to address the workers and answer their questions. It is found that the voluntary deductions are not popular, and that in most cases the employees prefer to have a foreman or fellow-worker as collector.

The villages presented naturally a quite different problem, and it was found that the best plan was to invite each village to form its own hospital aid committee, consisting of local collectors, with a local treasurer, secretary, and chairman. Someone is asked in each village to get up a meeting, at which a representative of the Radcliffe Infirmary explains the needs of the hospital, the details of the scheme, and its advantages to the working-classes.

Every local collector is provided with an official account-book, receipt-book, and a supply of cards. Each supporter has a card, on which the contributions are entered by the local collector, who pays sums collected to the local treasurer, to be remitted quarterly to the collector, at the Radcliffe Infirmary.

### THE ELASTICITY OF THE SCHEME.

On four points the rules are especially elastic :

1. No definite age-limit is fixed at which a contributor leaves off paying 1d. and begins to pay 2d. This, of course, is due to the fact that in different trades and occupations full wage-earning capacity is reached at a different age.
2. There is no qualifying period. In cases of accident or sudden illness a supporter after the payment of one 2d. contribution has the right to his free treatment, but where people join the scheme because they know they must shortly attend the hospital they are asked to pay at least a month's contribution in retrospect.
3. No limit of income is fixed above which anyone cannot become a supporter. The scheme, of course, is intended for the classes coming under the National Health Insurance Act mainly, but it was felt that hardships might be inflicted on people whose income was just above the £250 limit if they were excluded, and in the case of people who feel they ought to give more and can, they are allowed to pay an annual subscription—*e.g.*, of a guinea in advance on their cards, so that at the same time they secure the hospital benefit they may need. It is, however, always made quite plain that the scheme is one for those people only who cannot afford to pay for adequate medical treatment in their own homes.
4. No period is fixed at which a contributor loses his benefits through arrears, and it is found as a result that if contributors fall in arrears they generally pay up when applying to their local Treasurer for their admission form. In cases of poverty through ill-health or unemployment the Village Treasurer is allowed to use his discretion.

In fact, it is not found at present that the scheme suffers through the power given to the village committees to decide on any of these points. Some committees are forming a small fund out of which to pay arrears, *e.g.*, through destitution; and they all appear to appreciate the way in which the elasticity of the rules enables them to adapt the provisions of the scheme to individual circumstances. In this way "hard cases" are practically non-existent, while the committees are too keen to allow abuses. The Lady Almoner at Radcliffe Infirmary can always be referred to in the final resort; but her work in connection with the scheme consists largely in assessing the charges made for maintenance and treatment where patients have not yet come into it. It is also through the almoner's department that the committee receives large numbers of applications from people who are anxious for the scheme to be started in their respective villages.

### CO-ORDINATION WITH COTTAGE HOSPITALS.

A very important point in connection with the scheme has been that of co-ordination with the cottage hospitals in the district. After three conferences held at Oxford between their representatives and those of the Radcliffe Infirmary, the following resolutions were agreed to :

1. That the Radcliffe Infirmary should, by the organisation which it has now established in working order, organise the collection throughout the whole district, and that the contributory scheme be extended as far as possible, except where the population decide otherwise.
2. The distribution of funds collected under the contributory scheme from districts in which the interests of the local cottage hospitals are affected, shall be carried out half-yearly by an independent board, upon which shall be placed (1) representatives of the hospitals concerned; (2) elected representatives of the contributors; (3) a person experienced in hospital administration whose appointment shall be mutually agreed upon, and who shall act as arbitrator.

All the cottage hospitals have not yet put this agreement into operation, and three or four of them probably will prefer, at any rate for the present, not to do so.

Four of them, however, have come in, and in the case of Abingdon the Allocation Board is about to divide the proceeds of the first half-year's collections.

What the Scheme has Produced.

Turning to the results of the scheme, they are up to the present satisfactory. Three hundred and fourteen firms, six market towns, and 210 villages have joined since the scheme was started last September. For the quarter ending March 25 the collections amounted to £3,114 6s. 4d., and for the quarter ending June 24 they are now over £4,000, and the sum will be still higher, as all villages and firms have not yet sent in their contributions. Nearly 50,000 cards have now been issued, many of them, of course, bearing 4d. contributions. Considerably over 100 villages and many firms have still to come in, and steady progress is being made; by Christmas it is hoped that practically the whole ground will be covered. As a result, the Radcliffe has already been able to increase its activities and provide more efficiently for the needs of the district it serves. Another women's surgical ward has been opened, and it is hoped that another ward for men will be opened during the coming winter. A maternity home of sixteen beds, with an ante-natal clinic, the building for which had been bought and enlarged previously, has been equipped and opened; and the Committee of the hospital has also felt itself justified in accepting the gift of a house and 43 acres of ground on one of the hills surrounding Oxford, which will serve as a convalescent home. Optimism may surely at length be allowed on a modest scale, when after only nine months' work the contributions are coming in at the rate of over £16,000 a year.

